# *tert*-Butyl 4-isopropyl-2-oxo-6-phenyl-3,4-dihydro-2*H*-pyran-3-carboxyl­ate

**DOI:** 10.1107/S160053681001367X

**Published:** 2010-04-17

**Authors:** Wei Chen, Miao Yu, Si Li, Ning Jiao

**Affiliations:** a Chinese PLA Postgraduate Medical School, No. 28 Fuxing Road, Beijing 100853, People’s Republic of China; b Department of Radiology, Chinese PLA General Hospital, No. 28 Fuxing Road, Beijing 100853, People’s Republic of China; c State Key Laboratory of Natural and Biomimetic Drugs, School of Pharmaceutical Sciences, Peking University, Xue Yuan Rd 38, Beijing 100191, People’s Republic of China

## Abstract

In the title compound, C_19_H_24_O_4_, the six-membered lactone ring adopts an envelope conformation with the *tert*-butoxy­carbonyl and isopropyl substituents in axial positions, and the phenyl group in an equatorial position. In the crystal structure, weak inter­molecular C—H⋯O hydrogen bonds link the mol­ecules into centrosymmetric dimers.

## Related literature

For the applications and synthesis of endocyclic enol lactones, see: Davies & Jin (2004 ▶); Evans *et al.* (2005 ▶); Krafft & Katzenellenbogen (1981 ▶); Li *et al.* (2007 ▶); Zeni *et al.* (2004 ▶); Zhao *et al.* (1997 ▶); Jimenez-Tenorio *et al.* (2001 ▶). For the synthesis, see: Li *et al.* (2009 ▶).
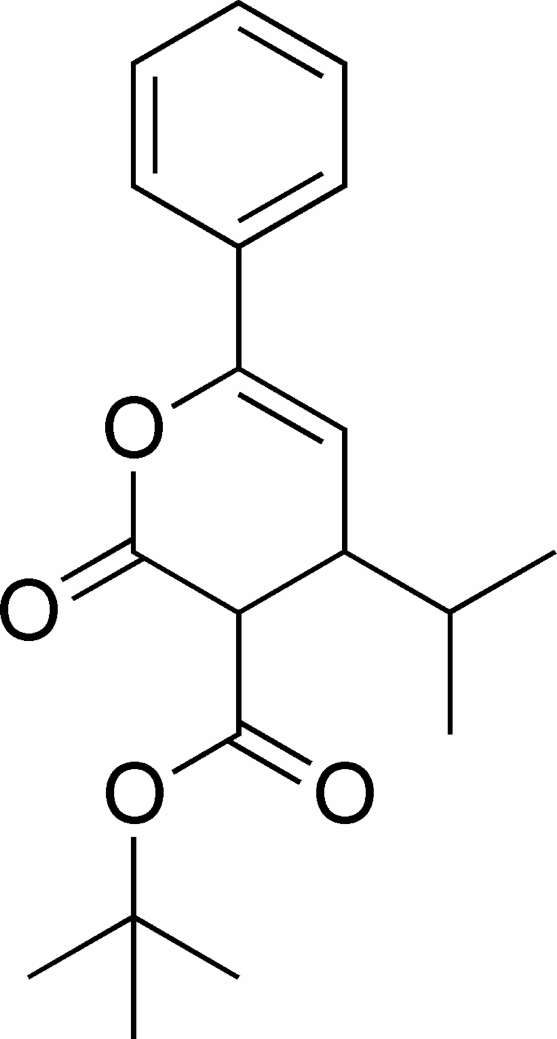


## Experimental

### 

#### Crystal data

C_19_H_24_O_4_
*M* *_r_* = 316.38
Triclinic, 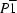

*a* = 8.6163 (9) Å
*b* = 10.888 (1) Å
*c* = 11.261 (1) Å
α = 68.393 (2)°
β = 79.118 (2)°
γ = 67.998 (2)°
*V* = 909.09 (15) Å^3^
*Z* = 2
Mo *K*α radiation
μ = 0.08 mm^−1^
*T* = 293 K
0.48 × 0.46 × 0.42 mm

#### Data collection

Bruker SMART CCD area-detector diffractometer
Absorption correction: multi-scan (*SADABS*; Sheldrick, 2004 ▶) *T*_min_ = 0.760, *T*_max_ = 1.000
4986 measured reflections
3510 independent reflections
2759 reflections with *I* > 2σ(*I*)
*R*_int_ = 0.051

#### Refinement

*R*[*F* ^2^ > 2σ(*F* ^2^)] = 0.055
*wR*(*F* ^2^) = 0.158
*S* = 1.04
3510 reflections
213 parameters
H-atom parameters constrained
Δρ_max_ = 0.22 e Å^−3^
Δρ_min_ = −0.21 e Å^−3^

### 

Data collection: *SMART* (Bruker, 2001 ▶); cell refinement: *SAINT* (Bruker, 2001 ▶); data reduction: *SHELXTL* (Sheldrick, 2008 ▶); program(s) used to solve structure: *SHELXS97* (Sheldrick, 2008 ▶); program(s) used to refine structure: *SHELXL97* (Sheldrick, 2008 ▶); molecular graphics: *ORTEP-3* (Farrugia, 1997 ▶) and *DIAMOND* (Brandenburg, 1998 ▶); software used to prepare material for publication: *SHELXL97*.

## Supplementary Material

Crystal structure: contains datablocks I, global. DOI: 10.1107/S160053681001367X/lx2139sup1.cif

Structure factors: contains datablocks I. DOI: 10.1107/S160053681001367X/lx2139Isup2.hkl

Additional supplementary materials:  crystallographic information; 3D view; checkCIF report

## Figures and Tables

**Table 1:** Hydrogen-bond geometry (Å, °)

*D*—H⋯*A*	*D*—H	H⋯*A*	*D*⋯*A*	*D*—H⋯*A*
C2—H2⋯O1^i^	0.98	2.44	3.407 (2)	170

Symmetry code: (i) 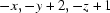.

**Fig. 1.**
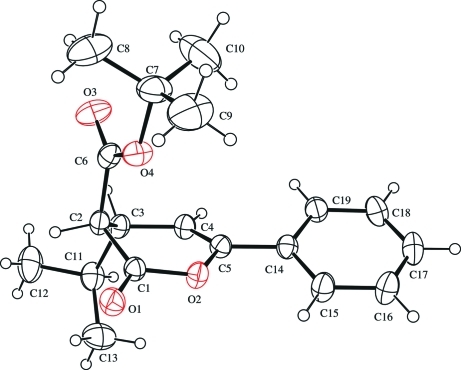
The molecular structure of the title compound with the atom numbering scheme. Displacement ellipsoids are drawn at the 30% probability level. H atoms are presented as a small spheres of arbitrary radius.

**Fig. 2.**
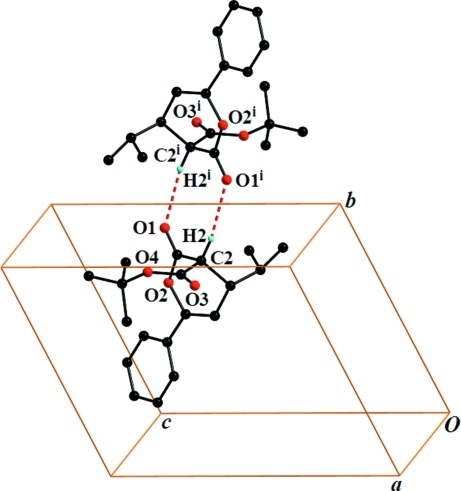
C—H···O interactions (dotted lines) in the crystal structure of the title compound. [Symmetry codes: (i) - x, - y + 2, - z + 1.]

## supplementary crystallographic information

### Comment

Endocyclic enol lactones are important structural elements of biologically active natural products (Zhao *et al.*, 1997) and useful synthetic intermediates for organic synthesis (Evans *et al.*, 2005, Davies *et al.*, 2004). The cyclization of alkynoic acids under acidic conditions (Krafft *et al.*, 1981) , employing transition-metal complexes as catalysts (Zeni *et al.*, 2004, Valerga *et al.*, 2001),and the carbonylation coupling of alkynes and 1,3-dicarbonyl compounds are main synthetic pathways for the preparation of Enol lactones (Li *et al.*, 2007)

In the title compound as shown in Fig. 1, the six-membered lactone ring adopts an envelope conformation with the tert-butoxycarbonyl, isopropyl and phenyl groups attached to it. The tert-butoxycarbonyl and isopropyl groups occupy axial positions, and the phenyl group occupies equatorial position. The crystal packing (Fig. 2) is stabilized by weak intermolecular C—H···O hydrogen bonds between the pyran H atom and the oxygen of the C═O unit in pyran ring, with a C2—H2···O1^i^ (Table 1).

### Experimental

The title compound was obtained as a by-product in the copper-catalyzed tandem conjugate addition–cyclization–hydrolysis–decarboxylation reactions of alkynes and 5-alkylidene-Meldrum's acids (Jiao *et al.*, 2009) acids as follows: To a mixture of CuBr (20 mg, 0.1 mmol), 1-ethynylbenzene (102 mg, 1 mmol) in H_2_O : t-BuOH = 10 : 1 (3 ml) was added and 2,2-dimethyl-5-(2-methylpropylidene)-1,3-dioxane-4,6-dione (99 mg, 0.5 mmol) at room temperature. The resulting mixture was refluxed for 10 h monitored by TLC. After evaporation, the residue was carefully purified by flash chromatography on silica gel. The title compound was obtained as a by-product (25% yield), which was crystallized from n-hexane-ethyl acetate.

### Refinement

All H atoms were positioned geometrically and refined using a riding model, with C—H = 0.93 Å for aryl, 0.98 Å for methyne and 0.96 Å for methyl H atoms. *U*_iso_(H) = 1.2*U*_eq_(C) for aryl and methyne H atoms, and 1.5*U*_eq_(C) for methyl H atoms.

### Crystal data

**Table:**

C_19_H_24_O_4_	*Z* = 2
*M**_r_* = 316.38	*F*(000) = 340
Triclinic, *P*1	*D*_x_ = 1.156 Mg m^−^^3^
Hall symbol: -P 1	Mo *K*α radiation, λ = 0.71073 Å
*a* = 8.6163 (9) Å	Cell parameters from 2216 reflections
*b* = 10.888 (1) Å	θ = 4.8–55.3°
*c* = 11.261 (1) Å	µ = 0.08 mm^−^^1^
α = 68.393 (2)°	*T* = 293 K
β = 79.118 (2)°	Prismatic, colorless
γ = 67.998 (2)°	0.48 × 0.46 × 0.42 mm
*V* = 909.09 (15) Å^3^	

### Data collection

**Table:**

Bruker SMART CCD area-detector diffractometer	3510 independent reflections
Radiation source: fine-focus sealed tube	2759 reflections with *I* > 2σ(*I*)
graphite	*R*_int_ = 0.051
Detector resolution: 10.0 pixels mm^-1^	θ_max_ = 26.0°, θ_min_ = 2.0°
φ and ω scans	*h* = −10→10
Absorption correction: multi-scan (*SADABS*; Sheldrick, 2004)	*k* = −10→13
*T*_min_ = 0.760, *T*_max_ = 1.000	*l* = −13→12
4986 measured reflections	

### Refinement

**Table:**

Refinement on *F*^2^	Primary atom site location: structure-invariant direct methods
Least-squares matrix: full	Secondary atom site location: difference Fourier map
*R*[*F*^2^ > 2σ(*F*^2^)] = 0.055	Hydrogen site location: inferred from neighbouring sites
*wR*(*F*^2^) = 0.158	H-atom parameters constrained
*S* = 1.03	*w* = 1/[σ^2^(*F*_o_^2^) + (0.0925*P*)^2^ + 0.0377*P*] where *P* = (*F*_o_^2^ + 2*F*_c_^2^)/3
3510 reflections	(Δ/σ)_max_ < 0.001
213 parameters	Δρ_max_ = 0.22 e Å^−^^3^
0 restraints	Δρ_min_ = −0.21 e Å^−^^3^

### Special details

**Table:**

Geometry. All esds (except the esd in the dihedral angle between two l.s. planes) are estimated using the full covariance matrix. The cell esds are taken into account individually in the estimation of esds in distances, angles and torsion angles; correlations between esds in cell parameters are only used when they are defined by crystal symmetry. An approximate (isotropic) treatment of cell esds is used for estimating esds involving l.s. planes.
Refinement. Refinement of F^2^ against ALL reflections. The weighted R-factor wR and goodness of fit S are based on F^2^, conventional R-factors R are based on F, with F set to zero for negative F^2^. The threshold expression of F^2^ > 2sigma(F^2^) is used only for calculating R-factors(gt) etc. and is not relevant to the choice of reflections for refinement. R-factors based on F^2^ are statistically about twice as large as those based on F, and R- factors based on ALL data will be even larger.

### Fractional atomic coordinates and isotropic or equivalent isotropic displacement parameters (Å^2^)

**Table:**

	*x*	*y*	*z*	*U*_iso_*/*U*_eq_	
O1	−0.04144 (14)	0.87317 (11)	0.66284 (11)	0.0535 (3)	
O2	0.07228 (13)	0.64576 (11)	0.71597 (10)	0.0461 (3)	
O3	0.48770 (16)	0.75686 (16)	0.51105 (14)	0.0721 (4)	
O4	0.33639 (14)	0.77581 (13)	0.69310 (12)	0.0554 (3)	
C1	0.06511 (19)	0.77682 (15)	0.63769 (14)	0.0408 (4)	
C2	0.19583 (19)	0.78720 (16)	0.52821 (14)	0.0425 (4)	
H2	0.1560	0.8805	0.4644	0.051*	
C3	0.2282 (2)	0.67697 (16)	0.46303 (15)	0.0441 (4)	
H3	0.3364	0.6694	0.4143	0.053*	
C4	0.2483 (2)	0.53947 (16)	0.56580 (15)	0.0450 (4)	
H4	0.3113	0.4581	0.5464	0.054*	
C5	0.18081 (19)	0.52752 (15)	0.68321 (15)	0.0412 (4)	
C6	0.3589 (2)	0.77079 (16)	0.57572 (16)	0.0470 (4)	
C7	0.4744 (3)	0.7625 (3)	0.7630 (2)	0.0739 (6)	
C8	0.5394 (3)	0.8833 (3)	0.6928 (3)	0.0982 (9)	
H8A	0.5896	0.8764	0.6108	0.147*	
H8B	0.6216	0.8799	0.7418	0.147*	
H8C	0.4481	0.9703	0.6812	0.147*	
C9	0.3851 (4)	0.7727 (4)	0.8901 (2)	0.1092 (10)	
H9A	0.2958	0.8610	0.8764	0.164*	
H9B	0.4630	0.7658	0.9448	0.164*	
H9C	0.3398	0.6978	0.9296	0.164*	
C10	0.6078 (4)	0.6211 (3)	0.7773 (4)	0.1282 (12)	
H10A	0.5556	0.5509	0.8000	0.192*	
H10B	0.6796	0.5992	0.8432	0.192*	
H10C	0.6730	0.6233	0.6979	0.192*	
C11	0.0973 (2)	0.7132 (2)	0.36787 (17)	0.0589 (5)	
H11	0.1194	0.6283	0.3467	0.071*	
C12	0.1187 (4)	0.8256 (3)	0.2436 (2)	0.0887 (8)	
H12A	0.0410	0.8410	0.1845	0.133*	
H12B	0.2312	0.7957	0.2076	0.133*	
H12C	0.0976	0.9111	0.2601	0.133*	
C13	−0.0809 (3)	0.7518 (3)	0.4235 (2)	0.0799 (6)	
H13A	−0.1104	0.8386	0.4398	0.120*	
H13B	−0.0909	0.6793	0.5021	0.120*	
H13C	−0.1549	0.7622	0.3639	0.120*	
C14	0.1969 (2)	0.39882 (16)	0.79280 (14)	0.0423 (4)	
C15	0.0804 (2)	0.39429 (19)	0.89627 (17)	0.0575 (5)	
H15	−0.0092	0.4750	0.8975	0.069*	
C16	0.0954 (3)	0.2717 (2)	0.99749 (19)	0.0686 (6)	
H16	0.0167	0.2701	1.0665	0.082*	
C17	0.2272 (3)	0.1519 (2)	0.99593 (19)	0.0678 (6)	
H17	0.2381	0.0692	1.0643	0.081*	
C18	0.3423 (3)	0.15430 (19)	0.89392 (19)	0.0637 (5)	
H18	0.4300	0.0726	0.8925	0.076*	
C19	0.3295 (2)	0.27597 (18)	0.79372 (17)	0.0531 (4)	
H19	0.4099	0.2766	0.7258	0.064*	

### Atomic displacement parameters (Å^2^)

**Table:**

	*U*^11^	*U*^22^	*U*^33^	*U*^12^	*U*^13^	*U*^23^
O1	0.0522 (7)	0.0401 (7)	0.0579 (7)	−0.0079 (5)	0.0052 (5)	−0.0165 (5)
O2	0.0517 (6)	0.0357 (6)	0.0438 (6)	−0.0129 (5)	0.0106 (5)	−0.0134 (5)
O3	0.0540 (8)	0.0982 (11)	0.0823 (10)	−0.0365 (7)	0.0214 (7)	−0.0508 (9)
O4	0.0482 (7)	0.0722 (9)	0.0523 (7)	−0.0250 (6)	0.0004 (5)	−0.0241 (6)
C1	0.0431 (8)	0.0347 (8)	0.0416 (8)	−0.0115 (7)	−0.0013 (6)	−0.0111 (6)
C2	0.0481 (9)	0.0342 (8)	0.0390 (8)	−0.0142 (7)	0.0015 (7)	−0.0068 (6)
C3	0.0499 (9)	0.0437 (9)	0.0368 (8)	−0.0174 (7)	0.0050 (7)	−0.0129 (7)
C4	0.0528 (9)	0.0372 (8)	0.0431 (9)	−0.0126 (7)	0.0014 (7)	−0.0156 (7)
C5	0.0432 (8)	0.0351 (8)	0.0437 (9)	−0.0113 (6)	0.0007 (6)	−0.0145 (7)
C6	0.0498 (9)	0.0401 (9)	0.0508 (10)	−0.0178 (7)	0.0057 (7)	−0.0159 (7)
C7	0.0590 (12)	0.1008 (17)	0.0697 (13)	−0.0321 (12)	−0.0105 (10)	−0.0277 (12)
C8	0.0908 (18)	0.139 (2)	0.108 (2)	−0.0700 (17)	0.0064 (15)	−0.0615 (18)
C9	0.110 (2)	0.184 (3)	0.0641 (15)	−0.076 (2)	−0.0044 (14)	−0.0459 (18)
C10	0.094 (2)	0.116 (3)	0.146 (3)	0.0030 (18)	−0.057 (2)	−0.026 (2)
C11	0.0786 (13)	0.0560 (11)	0.0459 (10)	−0.0254 (9)	−0.0091 (9)	−0.0156 (8)
C12	0.133 (2)	0.0810 (16)	0.0502 (12)	−0.0445 (15)	−0.0187 (13)	−0.0041 (11)
C13	0.0670 (13)	0.1032 (18)	0.0811 (15)	−0.0288 (12)	−0.0189 (11)	−0.0356 (13)
C14	0.0498 (9)	0.0409 (9)	0.0378 (8)	−0.0173 (7)	−0.0034 (7)	−0.0123 (7)
C15	0.0672 (11)	0.0478 (10)	0.0471 (10)	−0.0165 (8)	0.0072 (8)	−0.0120 (8)
C16	0.0870 (14)	0.0654 (13)	0.0459 (10)	−0.0332 (11)	0.0097 (10)	−0.0086 (9)
C17	0.0943 (15)	0.0496 (11)	0.0497 (11)	−0.0275 (11)	−0.0122 (10)	0.0020 (8)
C18	0.0752 (13)	0.0427 (10)	0.0580 (12)	−0.0073 (9)	−0.0148 (10)	−0.0071 (8)
C19	0.0539 (10)	0.0487 (10)	0.0482 (10)	−0.0123 (8)	−0.0030 (8)	−0.0112 (8)

### Geometric parameters (Å, °)

**Table:**

O1—C1	1.1927 (18)	C9—H9C	0.9600
O2—C1	1.3584 (18)	C10—H10A	0.9600
O2—C5	1.4091 (17)	C10—H10B	0.9600
O3—C6	1.198 (2)	C10—H10C	0.9600
O4—C6	1.318 (2)	C11—C13	1.508 (3)
O4—C7	1.482 (2)	C11—C12	1.517 (3)
C1—C2	1.507 (2)	C11—H11	0.9800
C2—C6	1.523 (2)	C12—H12A	0.9600
C2—C3	1.542 (2)	C12—H12B	0.9600
C2—H2	0.9800	C12—H12C	0.9600
C3—C4	1.489 (2)	C13—H13A	0.9600
C3—C11	1.545 (2)	C13—H13B	0.9600
C3—H3	0.9800	C13—H13C	0.9600
C4—C5	1.320 (2)	C14—C15	1.386 (2)
C4—H4	0.9300	C14—C19	1.394 (2)
C5—C14	1.467 (2)	C15—C16	1.380 (3)
C7—C10	1.510 (4)	C15—H15	0.9300
C7—C8	1.512 (4)	C16—C17	1.375 (3)
C7—C9	1.513 (3)	C16—H16	0.9300
C8—H8A	0.9600	C17—C18	1.368 (3)
C8—H8B	0.9600	C17—H17	0.9300
C8—H8C	0.9600	C18—C19	1.370 (2)
C9—H9A	0.9600	C18—H18	0.9300
C9—H9B	0.9600	C19—H19	0.9300
			
C1—O2—C5	120.35 (11)	H9B—C9—H9C	109.5
C6—O4—C7	122.54 (14)	C7—C10—H10A	109.5
O1—C1—O2	117.45 (14)	C7—C10—H10B	109.5
O1—C1—C2	125.81 (14)	H10A—C10—H10B	109.5
O2—C1—C2	116.73 (12)	C7—C10—H10C	109.5
C1—C2—C6	109.81 (13)	H10A—C10—H10C	109.5
C1—C2—C3	112.32 (12)	H10B—C10—H10C	109.5
C6—C2—C3	109.70 (13)	C13—C11—C12	111.18 (19)
C1—C2—H2	108.3	C13—C11—C3	113.38 (15)
C6—C2—H2	108.3	C12—C11—C3	111.50 (16)
C3—C2—H2	108.3	C13—C11—H11	106.8
C4—C3—C2	107.53 (12)	C12—C11—H11	106.8
C4—C3—C11	112.76 (13)	C3—C11—H11	106.8
C2—C3—C11	115.19 (14)	C11—C12—H12A	109.5
C4—C3—H3	107.0	C11—C12—H12B	109.5
C2—C3—H3	107.0	H12A—C12—H12B	109.5
C11—C3—H3	107.0	C11—C12—H12C	109.5
C5—C4—C3	123.13 (14)	H12A—C12—H12C	109.5
C5—C4—H4	118.4	H12B—C12—H12C	109.5
C3—C4—H4	118.4	C11—C13—H13A	109.5
C4—C5—O2	121.22 (13)	C11—C13—H13B	109.5
C4—C5—C14	127.91 (14)	H13A—C13—H13B	109.5
O2—C5—C14	110.81 (12)	C11—C13—H13C	109.5
O3—C6—O4	126.45 (17)	H13A—C13—H13C	109.5
O3—C6—C2	122.48 (16)	H13B—C13—H13C	109.5
O4—C6—C2	111.06 (13)	C15—C14—C19	118.15 (15)
O4—C7—C10	108.8 (2)	C15—C14—C5	121.59 (15)
O4—C7—C8	109.19 (19)	C19—C14—C5	120.24 (14)
C10—C7—C8	113.0 (2)	C16—C15—C14	120.91 (17)
O4—C7—C9	101.57 (16)	C16—C15—H15	119.5
C10—C7—C9	111.9 (2)	C14—C15—H15	119.5
C8—C7—C9	111.7 (2)	C17—C16—C15	119.78 (19)
C7—C8—H8A	109.5	C17—C16—H16	120.1
C7—C8—H8B	109.5	C15—C16—H16	120.1
H8A—C8—H8B	109.5	C18—C17—C16	120.00 (17)
C7—C8—H8C	109.5	C18—C17—H17	120.0
H8A—C8—H8C	109.5	C16—C17—H17	120.0
H8B—C8—H8C	109.5	C17—C18—C19	120.62 (18)
C7—C9—H9A	109.5	C17—C18—H18	119.7
C7—C9—H9B	109.5	C19—C18—H18	119.7
H9A—C9—H9B	109.5	C18—C19—C14	120.52 (17)
C7—C9—H9C	109.5	C18—C19—H19	119.7
H9A—C9—H9C	109.5	C14—C19—H19	119.7
			
C5—O2—C1—O1	172.04 (14)	C3—C2—C6—O4	−134.60 (13)
C5—O2—C1—C2	−9.4 (2)	C6—O4—C7—C10	−61.0 (3)
O1—C1—C2—C6	97.55 (18)	C6—O4—C7—C8	62.8 (2)
O2—C1—C2—C6	−80.92 (16)	C6—O4—C7—C9	−179.16 (19)
O1—C1—C2—C3	−140.10 (16)	C4—C3—C11—C13	72.2 (2)
O2—C1—C2—C3	41.43 (19)	C2—C3—C11—C13	−51.7 (2)
C1—C2—C3—C4	−47.02 (17)	C4—C3—C11—C12	−161.41 (17)
C6—C2—C3—C4	75.39 (15)	C2—C3—C11—C12	74.6 (2)
C1—C2—C3—C11	79.62 (17)	C4—C5—C14—C15	−158.33 (18)
C6—C2—C3—C11	−157.96 (13)	O2—C5—C14—C15	18.9 (2)
C2—C3—C4—C5	25.9 (2)	C4—C5—C14—C19	20.1 (3)
C11—C3—C4—C5	−102.21 (19)	O2—C5—C14—C19	−162.68 (14)
C3—C4—C5—O2	5.5 (2)	C19—C14—C15—C16	0.2 (3)
C3—C4—C5—C14	−177.58 (15)	C5—C14—C15—C16	178.69 (17)
C1—O2—C5—C4	−15.6 (2)	C14—C15—C16—C17	−0.3 (3)
C1—O2—C5—C14	166.99 (13)	C15—C16—C17—C18	−0.4 (3)
C7—O4—C6—O3	−0.8 (3)	C16—C17—C18—C19	1.2 (3)
C7—O4—C6—C2	179.96 (15)	C17—C18—C19—C14	−1.3 (3)
C1—C2—C6—O3	170.02 (16)	C15—C14—C19—C18	0.6 (3)
C3—C2—C6—O3	46.1 (2)	C5—C14—C19—C18	−177.89 (16)
C1—C2—C6—O4	−10.70 (17)		

### Hydrogen-bond geometry (Å, °)

**Table:**

*D*—H···*A*	*D*—H	H···*A*	*D*···*A*	*D*—H···*A*
C2—H2···O1^i^	0.98	2.44	3.407 (2)	170

Symmetry codes: (i) −*x*, −*y*+2, −*z*+1.
